# Evaluation of primers for the detection of deadwood-inhabiting archaea *via* amplicon sequencing

**DOI:** 10.7717/peerj.14567

**Published:** 2022-12-21

**Authors:** Julia Moll, Björn Hoppe

**Affiliations:** 1 Department of Soil Ecology, Helmholtz Centre for Environmental Research—UFZ, Halle (Saale), Germany; 2 Institute for National and International Plant Health, Julius Kühn Institute (JKI)—Federal Research Centre for Cultivated Plants, Braunschweig, Germany

**Keywords:** Archaea, Deadwood, Illumina sequencing, Primer, Methanobacteria

## Abstract

Archaea have been reported from deadwood of a few different tree species in temperate and boreal forest ecosystems in the past. However, while one of their functions is well linked to methane production any additional contribution to wood decomposition is not understood and underexplored which may be also attributed to lacking investigations on their diversity in this substrate. With this current work, we aim at encouraging further investigations by providing aid in primer choice for DNA metabarcoding using Illumina amplicon sequencing. We tested 16S primer pairs on genomic DNA extracted from woody tissue of four temperate deciduous tree species. Three primer pairs were specific to archaea and one prokaryotic primer pair theoretically amplifies both, bacterial and archaeal DNA. Methanobacteriales and Methanomassiliicoccales have been consistently identified as dominant orders across all datasets but significant variability in ASV richness was observed using different primer combinations. Nitrososphaerales have only been identified when using archaea-specific primer sets. In addition, the most commonly applied primer combination targeting prokaryotes in general yielded the lowest relative proportion of archaeal sequences per sample, which underlines the fact, that using target specific primers unraveled a yet unknown diversity of archaea in deadwood. Hence, archaea seem to be an important group of the deadwood-inhabiting community and further research is needed to explore their role during the decomposition process.

## Introduction

Deadwood has been demonstrated to be a valuable source of biodiversity in forest ecosystems (Lassauce et al., 2011; Stokland, Siitonen & Jonsson, 2012; Seibold et al., 2015), as it provides habitat and nutrients for various groups of organisms including fungi (Ódor et al., 2006; Kubartová et al., 2012; Hoppe et al., 2016; Yang et al., 2021), bacteria (Hoppe et al., 2015; Rinta-Kanto et al., 2016; Tláskal et al., 2017; Moll et al., 2018), arthropods (Wende et al., 2017; Doerfler et al., 2018; Müller et al., 2020) and, as most recently demonstrated nematodes (Moll et al., 2021b). In particular, fungi and bacteria have been reported to be highly specialized to their host trees (Moll et al., 2021a) and thus impact wood decomposition, either by utilizing wood as resource or by modifying its cellular structure (Clausen, 1996; Hatakka & Hammel, 2010). Hence, they are designated as main agents in this ecosystem process due to their complementary functions (Tláskal et al., 2021).

Archaea represent one of the three domains of live. They are highly diverse and occur in various environments, if not everywhere, and fulfill critical functions (Moissl-Eichinger et al., 2018). Their occurrence has been long linked to extreme environments under high temperatures, salt concentrations and extreme pH values such as hot springs, deep-sea sediments or salt marshes (Rothschild & Mancinelli, 2001; Korzhenkov et al., 2019). However, due to the working progress in culture-independent techniques during the last decades, many new archaeal lineages have been discovered and it became clear that they are more diverse than expected, ubiquitous distributed and associated to other microbes, plants and animals (Adam et al., 2017; Moissl-Eichinger et al., 2018). However, the role of archaea in wood still appears to be rather underexplored. To our best knowledge only three studies investigated archaea in deadwood reporting low but increasing abundances during wood decomposition (Rinta-Kanto et al., 2016; Pastorelli et al., 2021) and being restricted to specific wood substrates (Moll et al., 2018). The factors influencing their diversity, abundance and functioning are completely unknown, although some indications exist that wood-inhabiting archaea are linked to the production of methane. So-called methanogens produce methane as a metabolic byproduct during the degradation of organic compounds under low oxygen conditions, which makes heartwood of living and decomposing trees a suitable environment. Indeed, methane production from living trees, trunks and deadwood has already been reported (Covey et al., 2016; Pitz et al., 2018; Covey & Megonigal, 2019; Kipping et al., 2022). Nevertheless, the identity of wood-inhabiting methanogens and thus the underlying mechanisms of methane production are still unclear.

For PCR-based approaches, primer selection has a great effect on organismic diversity (Thijs et al., 2017; Karst et al., 2018). While bias during amplification cannot completely be avoided the right primer choice is known to improve the detection of target organisms, especially for groups usually associated with the co-amplification of non-target sequences, *e.g.*, arbuscular mycorrhizal fungi or archaea (Suzuki, Takahashi & Harada, 2020; Hathaway et al., 2021). A previous deadwood-based study discriminating between the outer and inner part of the wood observed a high proportion of methanogens in the heartwood of one (*Populus* sp.) of thirteen tree species using universal prokaryotic primers (Moll et al., 2018). In contrast, in the corresponding sapwood and in all other tree species archaea were not detected or only at very low abundances. Accordingly, with a similar approach Yip et al. (2019) identified a higher proportion of methanogens in heartwood in comparison to sapwood of the living *Populus deltoides*. In both studies the occurrence of this group was positively correlated with pH value.

This previous knowledge led us to the question, whether archaea are highly restricted to specific wood substrates or is their occurrence rather dependent on the applied approach. Hence, we tested four primer sets, three specific to archaea and one universal for prokaryotes for Illumina amplicon sequencing to compare the proportion of target sequences, the number of archaeal ASVs (amplicon sequence variants) and the number of archaeal orders. We hypothesized that an improved proportion of archaeal reads should result in a higher diversity revealing more archaeal groups showing that archaeal diversity in deadwood is not only limited to methanogens.

## Materials and Methods

### Deadwood samples

Deadwood samples were taken from the BELongDead (Biodiversity Exploratory Longterm Deadwood experiment) sampling campaign 2017 at the UNESCO Biosphere Reserve Schorfheide-Chorin (permit issued by responsible authority, Landesamt für Umwelt Brandenburg), 8 years after start of exposure (Fischer et al., 2010; Kahl et al., 2017). These wood samples were collected after bark removal in the form of chips using a cordless drill (Makita BDF 451) by driving the auger horizontally into the center of each log as previously described (Rieker et al., 2022). Previous analyses have shown a prominent ratio of archaeal sequences using the prokaryotic primer pair (515F/806R, Caporaso et al. (2011)) in specific deadwood samples, especially in logs of the genus *Populus* that showed a higher pH-value in comparison to other samples (Moll et al., 2018). Based on this prior knowledge, we selected 10 samples from four different tree species (2 × *Acer* spp., 2 × *Fagus sylvatica*, 2 × *Carpinus* *betulus* and 4 x *Populus* spp.) with a pH-value >5 to compare the traceability of archaea applying amplicon sequencing with different primer sets (Table 1).

**Table 1:** Source of wood sample.

Identifier	Tree name	Tree genus	pH
AH027	Maple	*Acer*	5.82
AH063	Maple	*Acer*	5.43
BU089	Beech	*Fagus*	6.33
BU100	Beech	*Fagus*	5.63
HBU067	Hornbeam	*Carpinus*	5.78
HBU079	Hornbeam	*Carpinus*	5.64
PA079	Aspen	*Populus*	6.53
PA084	Aspen	*Populus*	6.11
PA085	Aspen	*Populus*	6.05
PA066	Aspen	*Populus*	5.31

### DNA extraction, PCR and sequencing

Total genomic DNA was isolated from 0.1 g of each homogenized wood sample using the Quick-DNA Fecal/Soil Microbe Kit (Zymo Research, Irvine, CA, USA) according to the manufacturer’s protocol. Quality and quantity were checked using a NanoDrop spectrophotometer (Peqlab Biotechnologie GmbH, Erlangen, Germany). In order to compare archaeal diversity and relative ASV abundances four different primer systems were tested (Table 2). In each case, PCR was performed in 25 µl reactions, containing 12.5 µl of GoTaq Green Mastermix (Promega, Madison, Wisconsin, USA), 10 μM of each primer and 2 µl template DNA. If the quantity of the PCR product was too low, PCR was repeated with 3–4 µl template instead. Cycler conditions for archaeal primer sets were as follows: denaturation of 2 min at 95 °C followed by 35 cycles of 95 °C for 30 s, primer annealing for 30 s at 60 °C (Arch46 & Arch56) or 56 °C (Arch34), 72 °C for 45 s and a final elongation step at 72 °C for 5 min. PCR using the standard prokaryotic primers 515F/806R and 30 cycles was performed according to Moll et al. (2018). PCR products were purified, barcoded by Index PCR (Nextera XT Library Preparation Kit; Illumina, San Diego, CA, USA), again purified and pooled in equimolar amounts as described in (Moll et al., 2021b). The final library was sequenced using 2 × 300 bp paired end chemistry (MiSeq Reagent kit v3) on an Illumina MiSeq system (Illumina Inc., San Diego, CA, United States) at the Department of Soil Ecology of the Helmholtz Centre for Environmental Research–UFZ.

**Table 2:** Primer sequence information applied in this study; archaeal primers have been chosen based on a preselection of Fischer et al. (2016) suitable for next generation sequencing. The original citation for each respective primer is shown.

Pair	Name	Oligonucleotide sequence (5′-3′)	Reference
Prok	515F	GTGCCAGCMGCCGCGGTAA	Caporaso et al. (2011)
	806R	GGACTACHVGGGTWTCTAAT	Caporaso et al. (2011)
V34	Arch-0349F	GYGCASCAGKCGMGAAW	Takai & Horikoshi (2000)
	Arch-0806R	GGACTACVSGGGTATCTAAT	Takai & Horikoshi (2000)
V46	Arch-0519F	CAGCMGCCGCGGTAA	Ovreas et al. (1997)
	Arch-1041R	GGCCATGCACCWCCTCTC	Baker, Smith & Cowan (2003)
V56	Arch-0787F	ATTAGATACCCSBGTAGTCC	Yu et al. (2005)
	Arch-1043R	GCCATGCACCWCCTCT	Yu et al. (2005)

### Bioinformatics

The Dadasnake pipeline (Weißbecker, Schnabel & Heintz-Buschart, 2020), that combines the DADA2 workflow (Callahan et al., 2016) with several pre- and post-processing tools, was used for sequence data processing. Both primer sequences were cut using cutadapt v1.18 (Martin, 2011). Quality filtering slightly differed between primer sets according to the length of the amplified DNA fragment (Table S1). All obtained reads were then merged with an overlap of 12 bp and zero mismatches, expect for V46 allowing one mismatches for 20 bp overlap. For all primer sets chimera were removed based on the consensus algorithm and obtained ASVs (amplicon sequence variants) were taxonomically assigned using the Bayesian Classifier implemented in mothur (Schloss et al., 2009) against the prokaryotic SILVA database (SSU Ref, version 138) (Quast et al., 2013). The archaeal taxonomy was additionally predicted using the RDP classifier against the RDP 16S rRNA training set (No. 18 07/2020) (Cole et al., 2014) and this is finally reported. Due to the constant updates in their taxonomic classification especially at the phylum level (Rinke et al., 2021), we focused here on the order and genus level.

All raw reads have been submitted to the NCBI short read archive (SRA, https://www.ncbi.nlm.nih.gov/sra/) and are accessible under bioproject PRJNA862677. The four ASV tables are publicly available in the Biodiversity Exploratories Information System (https://www.bexis.uni-jena.de/ddm/data/Showdata/31300, https://www.bexis.uni-jena.de/ddm/data/Showdata/31301, https://www.bexis.uni-jena.de/ddm/data/Showdata/31302, https://www.bexis.uni-jena.de/ddm/data/Showdata/31303).

### Data analyses

Data were analysed using R 4.0.5 (R Core Team, 2020) and the ‘phyloseq’ and ‘microbiome’ packages (Lahti & Shetty, 2012–2019; McMurdie & Holmes, 2013). The loss of sequences during bioinformatics data processing was analyzed based on the ratio of remaining sequences after each step of the Dadasnake pipeline using the non-rarefied data sets. Afterwards, samples were rarified to 10,000 sequences to allow better comparison of archaeal diversity. Due to the co-amplification of larger fragments of non-target organisms for data set ArcV46 many reads have been lost already during merging which resulted in less than 10,000 sequences for several samples. As trade-off the data set ArcV46 was rarefied to 4,000 sequences, which still revealed saturated rarefaction curves (Fig. S1). These rarefied data sets were used to compare the proportion of archaeal orders and the number of observed archaeal ASVs between primer pairs. One archaeal order that was only observed once over all data sets represented by one ASV and only one sequence was removed due to uncertainties of its correctness and relevance. Plots were visualized using “ggplot2” (Wickham, 2016) and partly modified using CorelDRAW® Graphics Suite X8 (Corel Corporation, Ottawa, Canada).

## Results

### Sequence loss during data processing

**Figure 1:**
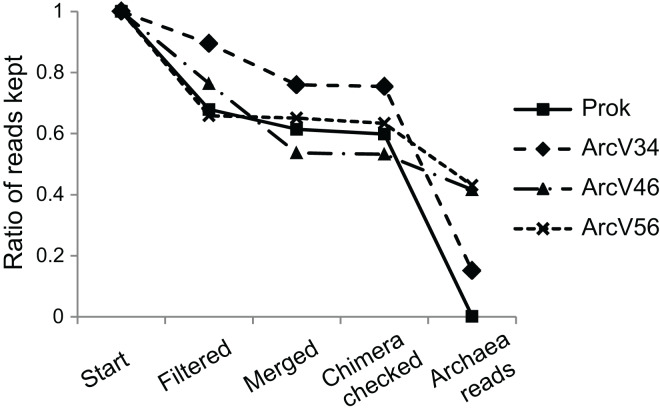
Remaining sequences during data processing (quality filtering, merging of forward and reverse read, chimera checking and taxonomical classification) for the four data sets (each primer combination is given in Table 2).

Four 16S primer sets have been compared, three being archaea specific and one prokaryotic primer pair amplifying both bacterial and archaeal DNA. After quality filtering more than 65% of all reads remained for all data sets (Fig. 1). Merging resulted only for minor read loss between 1–14% except for ArcV46 showing 23%. PCR verification *via* gelelectrophoresis revealed two band sizes, one around 600 bp, for several samples (BU100, AH0063, HBU072) of data set ArcV46. The missing overlap of the respective forward and reverse reads did probably not allow merging which explains the high number of lost reads during this step. Main differences between data sets have been observed during taxonomical assignment resulting in a huge loss of sequences that have not been affiliated to the Domain Archaea for ArcV34 and Prok data sets (15% and <1% archaeal sequences, respectively).

The application of universal prokaryotic primers (Prok data set) resulted in the lowest archaeal recovery of only 0.3% sequences and 5 ASVs (Table 3). Many sequences were of mitochondrial or chloroplast origin (Prok 13%, V34: 0%, V46: 0.2%, V56: 5%). The highest ratio of archaeal sequences was observed for the Arc.V56 data set (60.7% for the rarefied data). It also revealed the highest number of archaeal ASVs (44) and was the only data set that detected archaeal sequences in all samples (437 minimum archaeal sequences) (Table 3).

**Table 3:** Archaea sequence information of rarefied data sets (each primer combination is given in Table 2); all samples were rarefied to 10,000 sequences, except Arc.V46 was rarefied to 4,000 due to high sequence loss for several samples during merging.

	Total archaea reads	Min archaea reads	Max archaea reads	Average archaea reads	Ratio archaea reads (%)	Total ASVs	Archaea ASVs	Ratio archaea ASVs (%)
Prok	293	0	137	29.3	0.3	4,818	5	0.1
Arc.V34	18,393	0	8,200	1,839.3	18.4	1,485	16	1.1
Arc.V46	22,207	0	3,982	2,220.7	55.5	225	22	9.8
Arc.V56	60,706	437	9,979	6,070.6	60.7	732	44	6.1

### Taxonomic composition of archaea communities

**Figure 2:**
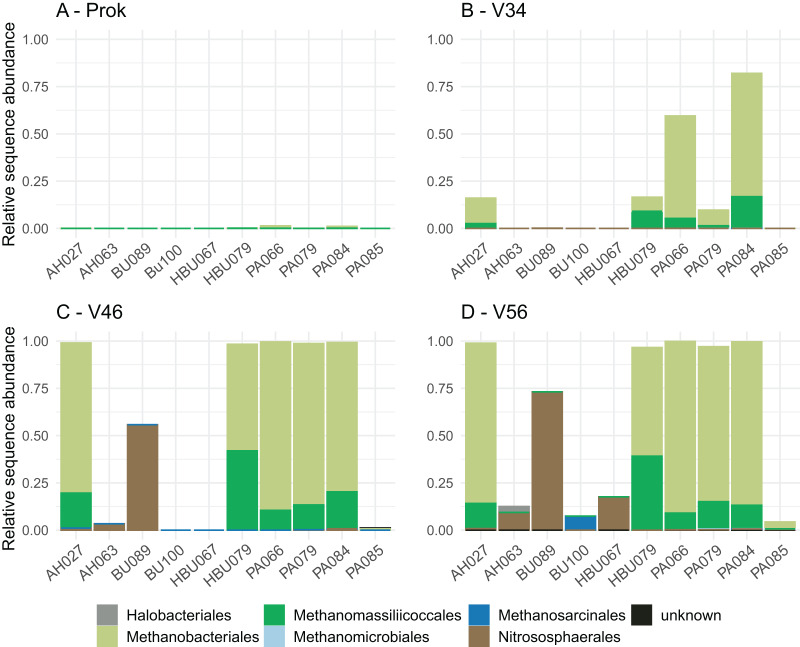
(A–D) Composition of archaeal orders observed in deadwood samples by four different primer sets (each primer combination is given in Table 2); AH, *Acer* spp.; BU, *Fagus sylvatica*; HBU, *Carpinus* *betulus *and PA, *Populus* spp.

**Figure 3:**
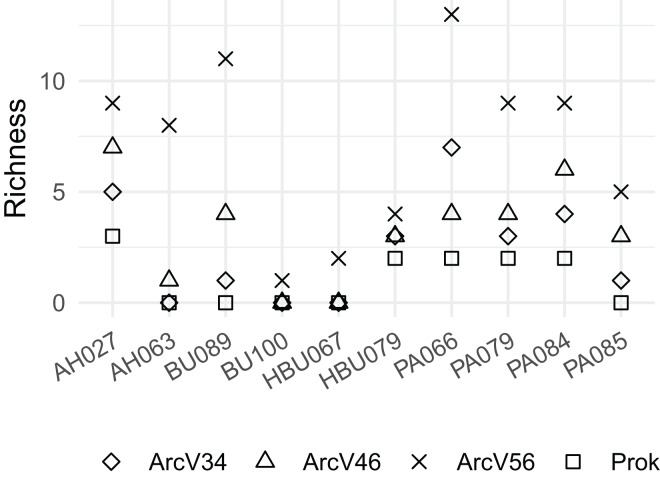
Observed number of archaeal ASVs for the different data sets (each primer combination is given in Table 2); AH, *Acer* spp.; BU, *Fagus sylvatica*; HBU, *Carpinus* *betulus* and PA, *Populus* spp.

Overall, four archaeal phyla have been observed. The most abundant phyla Euryarchaeota and Thermoplasmatota have been consistently identified with all primer pairs. Seven archaeal classes, eight orders, eleven families and eleven genera have been identified. The most abundant archaeal genera were *Methanobacterium* (Methanobacteriales) and Methanomassiliicoccus (Methanomassiliicoccales) independent of primer choice (Fig. 2). Archaeal ASV richness ranged from 0–13 archaeal ASVs per sample and strongly differed between data sets (Fig. 3). Despite a high range of observed ASV’s between wood samples, the use of primer set ArcV56 consistently revealed the highest number of archaeal ASVs for all samples (Fig. 3).

## Discussion

The present amplicon sequencing analysis demonstrates that archaea are present in decaying wood and their richness, the relative abundances of ASVs and their taxonomic diversity strongly depends on the chosen primer system.

The matrix from which DNA is isolated, namely the presence of DNA from other biota, influences the recovery of target organisms. This is known for PCR-based analyses of many groups, *e.g.*, arbuscular mycorrhizal fungi, metazoans or prokaryotes (Kohout et al., 2014; Beckers et al., 2016; Collins et al., 2019). For plant-associated microbial communities the co-amplification of chloroplasts and mitochondria is often challenging and potentially leads to huge loss of sequences, while the yield in other matrices are not as affected (Arenz et al., 2015; Beckers et al., 2016). In deadwood the use of universal prokaryotic primer has shown high amounts of such non-target sequences (Moll et al., 2018). This was confirmed here, showing that 8 years after tree death chloroplasts and mitochondria still accounted for 13% of sequences for the Prok data set. The presence of sufficient nucleotide mismatches against non-target DNA plays an important role, but the development of respective primers cannot be validated for all matrices, *i.e.*, for all potentially co-occurring biota. Hence, for the identification of organisms in new fields primers need to be tested and evaluated to ensure enough recovery of the targeted community. The primer pair V34 has already been applied for the investigation of archaeal communities in *Picea abies* deadwood samples (Rinta-Kanto et al., 2016). Indeed, several archaea have been identified, but authors mentioned the low yield of archaeal sequences and the strong differences between samples. Thus, they concluded that it is likely that only the most abundant groups have been detected which did not cover the entire archaeal diversity (Rinta-Kanto et al., 2016), which is in line with our findings.

Primer choice is not only known to greatly affect the amplification success of the targeted group but also its composition, which was also observed in the present study. The archaeal groups Methanobacteriales and Methanomassiliicoccales have been consistently identified as most dominant orders for all data sets. Methanogens have been previously identified in stems of living trees, especially that of *Populus* spp. (Yip et al., 2019). These findings are particularly of interest as the contribution of trees to global methane emission is a matter of debate and the identification of respective methane producers is an important puzzle piece to better understand CH_4_-producing mechanisms (Barba et al., 2019; Covey & Megonigal, 2019). A previous study detected high ratios of methanogens with 28% averaged sequence abundance of the microbial community in *Populus*-deadwood after 6 years of decay using the same universal 16S primers as applied here (Moll et al., 2018). Interestingly, results were very similar to that of Yip et al. (2019) constantly showing higher abundances in heartwood than in sapwood indicating more favorable conditions for these obligate anaerobic microbes. Here, we were also able to identify methanogens in deadwood even after 8 years of decomposition, albeit to lower extend, but not only for *Populus*. However, in contrast to Moll et al. (2018) and Yip et al. (2019) a composite wood sample including both, sapwood and heartwood have been used which likely contribute to the lower ratio of archaea. In addition, longer time since tree death can also serve as an explanation for the lower ratio, as CH_4_-emission based on methanogenic archaea is expected to be highly abundant in living trees and to decrease during decomposition after tree death (Covey & Megonigal, 2019). In contrast to Methanobacteriales and Methanomassiliicoccales, the order Nitrososphaerales have been identified only using the archaea specific primer sets (V34, V46, V56) (Fig. 2). This group of putative ammonia oxidizers (AOA) fulfill the first step of nitrification by converting ammonium to nitrite (Stein & Klotz, 2016). The utilized ammonium that has already been detected in the deadwood logs of the BELongDead experiment can potentially be provided by nitrogen fixation or nitrite reducing microorganisms. Although Nitrosophaerales dominated three samples, that did not show high methanogen abundances, the presence of both groups in deadwood is interesting, especially as methanogens, *i.e.*, members of the Methanobacteriales, are known for their ability to fix nitrogen (Leigh, 2000). Further studies are needed to unravel the interplay between methanogens, ammonium-oxidizers, nitrogen-fixing microorganisms or methanotrophs, that all seems to be a substantial part of the deadwood-inhabiting community (Hoppe et al., 2014; Mäkipää et al., 2018).

## Conclusions

In summary, for the deadwood samples of the tree species investigated (*Acer*, *Fagus*, *Carpinus* and *Populus*) the application of archaea-specific primers increases the recovery of archaeal sequences, the number of archaeal ASVs and taxonomic diversity. Hence, this study shows that an investigation of archaea by a separate amplicon library is recommended for deadwood as matrix. Both ArcV46 and ArcV56 exhibit more than 40% archaeal sequences. However, ArcV46 lost a high ratio of sequences for some samples during merging likely due to the co-amplification of a longer fragment making it difficult to include these specific samples for further analyses. ArcV56 was the only data set that detected archaeal ASVs consistently in all samples and the highest number of archaeal ASVs for all samples and the most comprehensive view of taxonomic diversity was observed. Despite shorter fragment lengths for ArcV56, taxonomic resolution was not reduced compared to ArcV46. In contrast, the shortness of the fragments made a truncation of reads possible. Due to the general decrease in quality with increasing read length this step usually supports read quality and hence facilitates data processing to reach sufficient sequence depth for diversity analyses. Ecologically, little is known about archaea in deadwood but they seem to be part of the wood-inhabiting community and potentially fulfill important biochemical functions. Future research on the identity of archaea in deadwood, their presence across tree species, drivers of their community combined with physiological tests is needed to provide an understanding of their diversity and activity in the deadwood system.

## Supplemental Information

10.7717/peerj.14567/supp-1 Supplemental Information 1 Quality filtering parameters during data processing using DADA2 for all respective primer sets. Trunc length: Truncate reads after respective bases, shorter reads are discarded. Trunc quality: Truncate reads after first bases less than or equal

10.7717/peerj.14567/supp-2 Supplemental Information 2 Rarefaction curves for all rarified datasets: (A) Prok, (B) V34, (C) V46 and (D) V56.

## Additional Information and Declarations

### Field Study Permissions

The following information was supplied relating to field study approvals (*i.e*., approving body and any reference numbers): Field experiments were approved by the responsible authority Landesamt für Umwelt Brandenburg (Ref. No. LfU_N1-4743h26+1#2126412017).

### DNA Deposition

The following information was supplied regarding the deposition of DNA sequences: All raw reads are available at the NCBI Short Read Archive (SRA): PRJNA862677.

### Data Availability

The following information was supplied regarding data availability: The four ASV tables are available in the Biodiversity Exploratories Information System: -https://www.bexis.uni-jena.de/ddm/data/Showdata/31300. -https://www.bexis.uni-jena.de/ddm/data/Showdata/31301. -https://www.bexis.uni-jena.de/ddm/data/Showdata/31302. -https://www.bexis.uni-jena.de/ddm/data/Showdata/31303.
