# Supplementary material for: Evaluation of primers for the detection of deadwood-inhabiting archaea via amplicon sequencing
Source: PeerJ. 2022 Dec 21;10:e14567. doi: 10.7717/peerj.14567 (PMC9789694; doi:10.7717/peerj.14567)
Supplement: Supplemental Information 1 — Trunc length: Truncate reads after respective bases, shorter reads are discarded. Trunc quality: Truncate reads after first bases less than or equal [file peerj-10-14567-s001.docx]

# Supplemental material

Table S1: Quality filtering parameters during data processing using DADA2 for all respective primer sets; Trunc length: Truncate reads after respective bases, shorter reads are discarded. Trunc quality: Truncate reads after first bases less than or equal to respective quality score, maxEE: Reads showing higher "expected errors" than maxEE will be removed, for: forward reads, rev: reverse reads.

| Quality parameters | Prok for/rev | V34 for/rev | V46 for/rev | V56 for/rev |
| --- | --- | --- | --- | --- |
| Trunc length [bp] | 170/ 130 | 0/ 0 | 0/ 0 | 190/ 130 |
| Trunc quality | 12, 12 | 10, 10 | 10, 10 | 12, 12 |
| maxEE | 0.5/ 0.5 | 2/ 2 | 2/ 2 | 0.5/ 0.5 |
