# Supplementary figures and images for: Evaluation of primers for the detection of deadwood-inhabiting archaea via amplicon sequencing
Source: PeerJ. 2022 Dec 21;10:e14567. doi: 10.7717/peerj.14567 (PMC9789694; doi:10.7717/peerj.14567)

**A** Prok 10,000 sequences

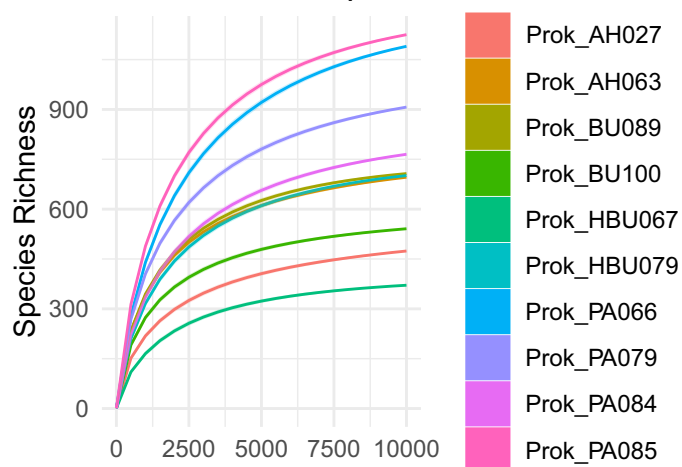

**B** V34 10,000 sequences

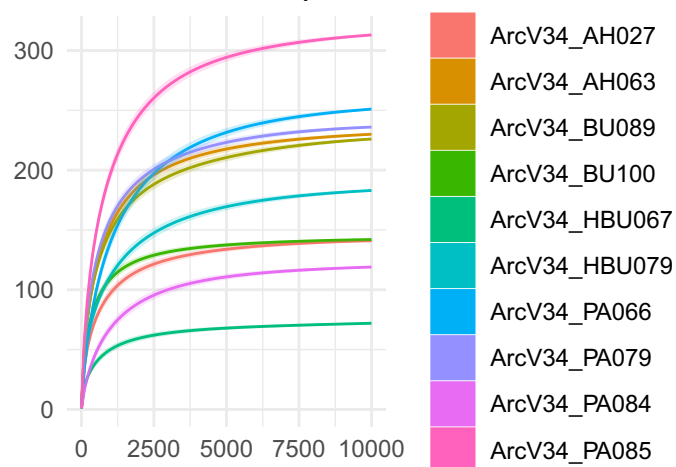

**C** V46 4,000 sequences

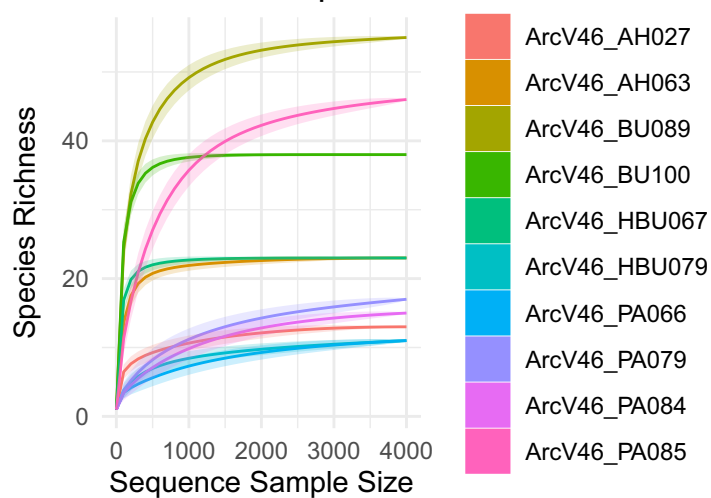

**D** V56 10,000 sequences

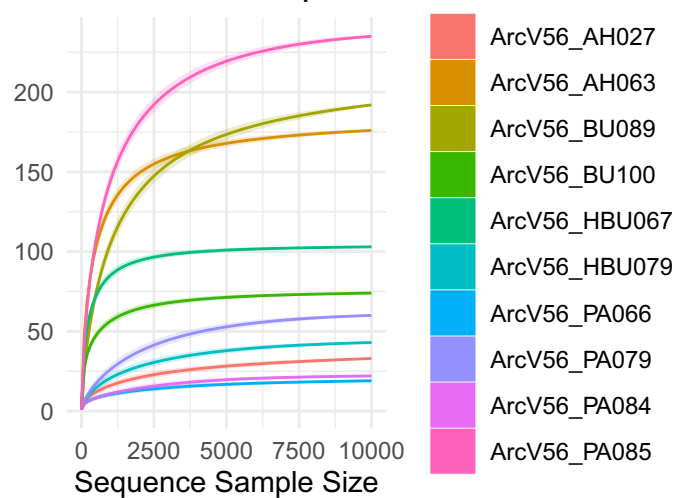

Supplement: Supplemental Information 2 [file peerj-10-14567-s002.pdf]
